# Supplementary material for: Pattern of Expression of Genes Involved in Systemic Inflammation and Glutathione Metabolism Reveals Exacerbation of COPD
Source: Antioxidants (Basel). 2024 Aug 6;13(8):953. doi: 10.3390/antiox13080953 (PMC11351727; doi:10.3390/antiox13080953)
Supplement: Supplementary file 1 [file antioxidants-13-00953-s001.zip › Supplementary Figure S1 text IOW.pdf]

Pattern of Expression of Genes Involved in Systemic Inflamma-tion and Glutathione Metabolism Reveals Exacerbation of COPD

Ingrid Oit-Wiscombe [1,2,3], László Virág [4,5], Kalle Kilk [2,3], Ursel Soomets [2,3] and Alan Altraja [1,6,*]

Institution:

1- Department of Pulmonology, University of Tartu, 50406 Tartu, Estonia

2- Institute of Biomedicine and Translational Medicine, University of Tartu, 50411 Tartu, Estonia; kalle.kilk@ut.ee (K.K.)

3- Centre of Excellence for Genomics and Translational Medicine, University of Tartu, 50411 Tartu, Estonia

4- Department of Medical Chemistry, Faculty of Medicine, University of Debrecen, 4032 Debrecen, Hungary; lvirag@med.unideb.hu

5- HUN-REN-DE Cell Biology and Signaling Research Group, 4032 Debrecen, Hungary

6- Lung Clinic, Tartu University Hospital, 50406 Tartu, Estonia

* Correspondence: alan.altraja@ut.ee

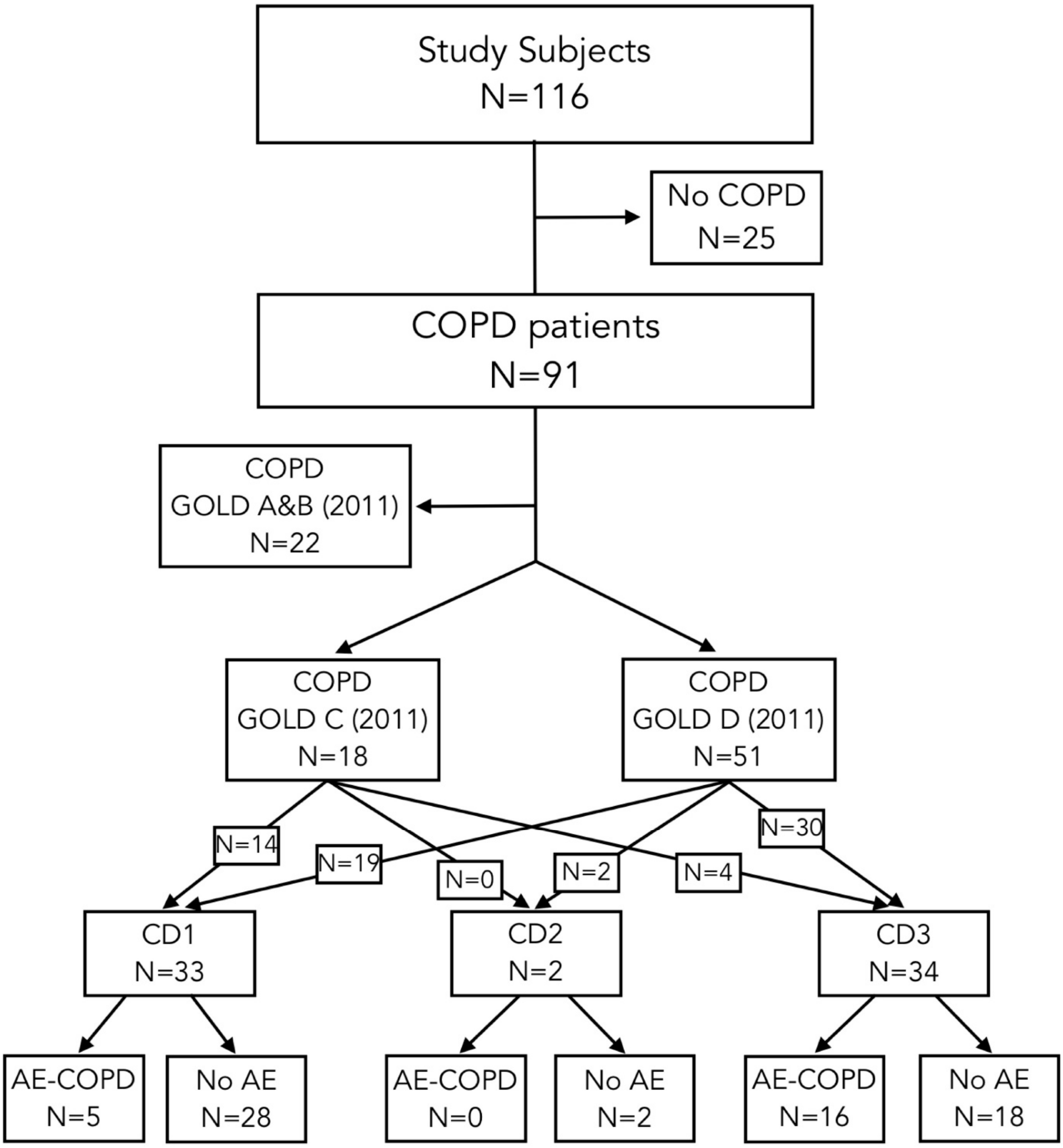
Study Subjects
N=116
No COPD
N=25
COPD patients
N=91
COPD
GOLD A&B (2011)
N=22
COPD
GOLD C (2011)
N=18
COPD
GOLD D (2011)
N=51
N=14
N=19
N=0
N=2
N=4
N=30
CD1
N=33
CD2
N=2
CD3
N=34
AE-COPD
N=5
No AE
N=28
AE-COPD
N=0
No AE
N=2
AE-COPD
N=16
No AE
N=18

Supplementary Figure S1. Flow diagram of patient distribution to chronic obstructive pulmonary disease (COPD) categories C and D according to the COPD GOLD 2011 classification [1] and sub-division to subgroups 1-3 according to Lange et. al. [2] (Patients diagnosed as having COPD divided into 3 groups, patients with forced expiratory flow in one second (FEV_1) <50% predicted and fewer than two exacerbations in the previous year, patients with FEV_1 ≥50% predicted and two or more exacerbations in the previous year, and patients with FEV_1 <50% predicted and two or more exacerbations in the previous year according to the Global Initiative for COPD consensus document 2011 (GOLD 2011) [1,2]).

1 Lange P, Marott JL, Vestbo J, et al. Prediction of the clinical course of chronic obstructive pulmonary disease, using the new GOLD classification: a study of the general population. Am J Respir Crit Care Med. 2012 Nov 15;186(10):975-81.

2 Global Initiative for Chronic Obstructive Lung Disease (GOLD). Global Strategy for the Diagnosis, Management, and Prevention of Chronic Obstructive Pulmonary Disease. 2011. Available from: https://goldcopd.org/
