# Supplementary material for: Pattern of Expression of Genes Involved in Systemic Inflammation and Glutathione Metabolism Reveals Exacerbation of COPD
Source: Antioxidants (Basel). 2024 Aug 6;13(8):953. doi: 10.3390/antiox13080953 (PMC11351727; doi:10.3390/antiox13080953)
Supplement: Supplementary file 1 [file antioxidants-13-00953-s001.zip › SupplementaryTables IOW.pdf]

Pattern of Expression of Genes Involved in Systemic Inflamma-tion and Glutathione Metabolism Reveals Exacerbation of COPD

Ingrid Oit-Wiscombe [1,2,3], László Virág [4,5], Kalle Kilk [2,3], Ursel Soomets [2,3] and Alan Altraja [1,6,*]

Institution:

1- Department of Pulmonology, University of Tartu, 50406 Tartu, Estonia

2- Institute of Biomedicine and Translational Medicine, University of Tartu, 50411 Tartu, Estonia; kalle.kilk@ut.ee (K.K.)

3- Centre of Excellence for Genomics and Translational Medicine, University of Tartu, 50411 Tartu, Estonia

4- Department of Medical Chemistry, Faculty of Medicine, University of Debrecen, 4032 Debrecen, Hungary; lvirag@med.unideb.hu

5- HUN-REN-DE Cell Biology and Signaling Research Group, 4032 Debrecen, Hungary

6- Lung Clinic, Tartu University Hospital, 50406 Tartu, Estonia

* Correspondence: alan.altraja@ut.ee

Supplementary table S1. Differences in the levels of expression of mRNA of enzymes involved in glutathione (GSH) metabolism and inflammation analysed by multinominal logistic regression. Odds ratios vs. comparators with 95% confidence intervals for each enzyme are shown. Data are adjusted to age, gender and body-mass index (BMI). a) Patients with chronic obstructive pulmonary disease (COPD) by severity of airflow limitation according to the Global Initiative for Chronic Obstructive Lung Disease (GOLD) [1] groups 1-4 compared to all non-obstructive individuals irrespective of their smoking history. b) Non-obstructive smokers and patients with COPD by airflow limitation severity according to GOLD groups 1-4 [1] compared to non-obstructive non-smokers. c) Patients with COPD according to the GOLD groups A-D [1] compared to pooled non-obstructive individuals irrespective of their smoking history. d) Patients with a history of at least two moderate or at least one severe exacerbation within the last year compared to all individuals, who have not experienced an exacerbation (non-obstructive non-smokers, non-obstructive smokers and patients with COPD by airflow limitation severity according to GOLD groups 1-4 without COPD exacerbation [1]).

	Group	Enzyme	OR	95% CI	p-value
a)	Reference group: all non-obstructive individuals irrespective of their smoking history				
	GOLD 1&2	5-LO	0.986	0.923-1.053	0.674
	GOLD 3		0.978	0.918-1.042	0.491
	GOLD 4		1.004	0.946-1.066	0.890
	GOLD 1&2	SOD1	0.984	0.931-1.041	0.578
	GOLD 3		1.017	0.97-1.067	0.489
	GOLD 4		1.022	0.974-1.073	0.377
	GOLD 1&2	PARP-1	49.685	18.731-131.795	<0.001
	GOLD 3		28.331	8.988-89.299	<0.001
	GOLD 4		1.232	0.414-3.664	0.708
	GOLD 1&2	LTA4H	1.039	0.889-1.214	0.631
	GOLD 3		1.088	0.959-1.234	0.189
	GOLD 4		0.947	0.832-1.078	0.413
	GOLD 1&2	HDAC2	0.406	0.293-0.562	<0.001
	GOLD 3		0.733	0.569-0.944	0.016
	GOLD 4		1.347	1.052-1.725	0.018
	GOLD 1&2	GSS	0.899	0.745-1.085	0.269
	GOLD 3		0.994	0.825-1.198	0.953
	GOLD 4		0.904	0.687-1.19	0.473

GOLD 1&2	GSR	1.035	0.973-1.101	0.272
GOLD 3		1.008	0.951-1.069	0.779
GOLD 4		1.030	0.971-1.093	0.328
GOLD 1&2	GPX	1.004	0.989-1.019	0.611
GOLD 3		1.001	0.987-1.015	0.894
GOLD 4		1.001	0.987-1.016	0.856
GOLD 1&2	GCLM	5.868	2.752-12.511	<0.001
GOLD 3		7.578	0.969-59.282	0.054
GOLD 4		63.472	5.05-797.832	0.001
GOLD 1&2	GCLC	0.414	0.304-0.564	<0.001
GOLD 3		0.710	0.553-0.911	0.007
GOLD 4		0.744	0.575-0.961	0.024
GOLD 1&2	DPP4	1.141	0.601-2.166	0.686
GOLD 3		0.835	0.618-1.128	0.241
GOLD 4		0.407	0.215-0.771	0.006
GOLD 1&2	COX2	1.113	0.963-1.286	0.147
GOLD 3		1.095	0.945-1.269	0.226
GOLD 4		0.937	0.794-1.105	0.440
b) Reference group: all non-obstructive non-smokers				
Non-obstructive smokers	5-LO	0.994	0.923-1.071	0.883
GOLD 1&2		0.979	0.909-1.055	0.585
GOLD 3		0.971	0.903-1.044	0.424
GOLD 4		0.997	0.93-1.07	0.939
Non-obstructive smokers	SOD1	0.985	0.922-1.053	0.654
GOLD 1&2		0.977	0.917-1.041	0.475
GOLD 3		1.010	0.953-1.07	0.745
GOLD 4		1.014	0.957-1.075	0.633
Non-obstructive smokers	PARP-1	0.442	0.104-1.879	0.269
GOLD 1&2		21.786	5.554-85.464	<0.001
GOLD 3		12.643	5.825-27.44	<0.001
GOLD 4		0.562	0.353-0.897	0.016
Non-obstructive smokers	LTA4H	0.836	0.714-0.98	0.027

GOLD 1&2		0.920	0.771-1.099	0.359
GOLD 3		0.967	0.833-1.122	0.658
GOLD 4		0.841	0.722-0.98	0.027
Non-obstructive smokers	HDAC2	1.112	0.842-1.468	0.454
GOLD 1&2		0.442	0.326-0.601	<0.001
GOLD 3		0.798	0.612-1.04	0.094
GOLD 4		1.451	1.105-1.906	0.007
Non-obstructive smokers	GSS	0.919	0.605-1.396	0.693
GOLD 1&2		0.882	0.705-1.103	0.270
GOLD 3		0.983	0.785-1.232	0.885
GOLD 4		0.895	0.707-1.133	0.357
Non-obstructive smokers	GSR	1.211	1.072-1.368	0.002
GOLD 1&2		1.217	1.077-1.374	0.002
GOLD 3		1.183	1.048-1.335	0.007
GOLD 4		1.212	1.073-1.368	0.002
Non-obstructive smokers	GPX	1.006	0.984-1.028	0.586
GOLD 1&2		1.008	0.987-1.03	0.452
GOLD 3		1.005	0.984-1.027	0.624
GOLD 4		1.006	0.985-1.027	0.594
Non-obstructive smokers	GCLM	3.405	0.524-22.14	0.200
GOLD 1&2		21.587	6.541-71.243	<0.001
GOLD 3		26.282	3.953-174.762	<0.001
GOLD 4		239.888	25.673-2241.521	<0.001
Non-obstructive smokers	GCLC	0.960	0.722-1.277	0.779
GOLD 1&2		0.402	0.287-0.561	<0.001
GOLD 3		0.703	0.532-0.929	0.013
GOLD 4		0.734	0.569-0.948	0.018
Non-obstructive smokers	DPP4	1.470	0.797-2.711	0.217
GOLD 1&2		1.430	0.773-2.648	0.254
GOLD 3		1.028	0.726-1.455	0.877
GOLD 4		0.502	0.315-0.8	0.004
Non-obstructive smokers	COX2	1.040	0.818-1.321	0.749

GOLD 1&2		1.164	0.971-1.395	0.100
GOLD 3		1.146	0.965-1.362	0.121
GOLD 4		0.976	0.819-1.163	0.784

c) Reference group: all non-obstructive individuals irrespective of their smoking history

GOLD A	5-LO	1.007	0.939-1.079	0.850
GOLD B		1.011	0.948-1.079	0.737
GOLD C		1.001	0.938-1.067	0.986
GOLD D		0.998	0.932-1.069	0.965
GOLD A	SOD1	1.037	0.981-1.097	0.202
GOLD B		1.011	0.955-1.07	0.707
GOLD C		1.021	0.966-1.079	0.464
GOLD D		1.014	0.957-1.073	0.643
GOLD A	PARP-1	20.747	6.26-68.757	<0.001
GOLD B		9.251	4.836-17.696	<0.001
GOLD C		23.108	8.534-62.568	<0.001
GOLD D		23.359	7.574-72.038	<0.001
GOLD A	LTA4H	1.229	1.064-1.42	0.005
GOLD B		0.938	0.815-1.081	0.376
GOLD C		1.122	0.984-1.281	0.086
GOLD D		1.115	0.972-1.28	0.120
GOLD A	HDAC2	0.565	0.427-0.747	<0.001
GOLD B		0.593	0.423-0.832	0.002
GOLD C		0.841	0.634-1.115	0.228
GOLD D		1.029	0.759-1.396	0.855
GOLD A	GSS	0.852	0.667-1.088	0.199
GOLD B		0.818	0.606-1.104	0.188
GOLD C		0.906	0.679-1.208	0.500
GOLD D		0.934	0.677-1.288	0.677
GOLD A	GSR	1.011	0.95-1.075	0.734
GOLD B		1.017	0.957-1.08	0.590
GOLD C		1.016	0.959-1.075	0.594
GOLD D		1.021	0.963-1.083	0.479

GOLD A	GPX	1.003	0.988-1.018	0.737
GOLD B		1.002	0.987-1.017	0.830
GOLD C		1.002	0.988-1.016	0.818
GOLD D		1.001	0.986-1.016	0.877
GOLD A	GCLM	29.361	2.133-404.152	0.012
GOLD B		107.541	2.017-5733.287	0.021
GOLD C		55.510	2.251-1368.682	0.014
GOLD D		81.576	2.287-2909.528	0.016
GOLD A	GCLC	0.359	0.269-0.48	0.000
GOLD B		0.799	0.544-1.174	0.252
GOLD C		0.581	0.424-0.796	<0.001
GOLD D		0.714	0.504-1.011	0.058
GOLD A	DPP4	1.121	0.538-2.336	0.760
GOLD B		0.374	0.219-0.639	<0.001
GOLD C		0.554	0.27-1.138	0.108
GOLD D		0.369	0.16-0.852	0.020
GOLD A	COX2	1.093	0.941-1.27	0.244
GOLD B		0.947	0.81-1.106	0.490
GOLD C		1.018	0.878-1.181	0.811
GOLD D		0.993	0.838-1.176	0.931

d) Reference group: non-obstructive non-smokers, non-obstructive smokers and patients with COPD by airflow limitation severity according to GOLD groups 1-4 without COPD exacerbation

Mild & moderate	5-LO	0.998	0.929-1.072	0.956
Severe		0.993	0.927-1.064	0.841
Very severe		1.018	0.954-1.087	0.587
Mild & moderate	SOD1	0.991	0.932-1.054	0.770
Severe		1.019	0.962-1.079	0.521
Very severe		1.028	0.97-1.088	0.354
Mild & moderate	PARP-1	53.447	15.212-187.79	<0.001
Severe		26.351	10.736-64.68	<0.001
Very severe		1.216	0.515-2.871	0.655
Mild & moderate	LTA4H	1.055	0.897-1.242	0.516

Severe		1.117	0.98-1.272	0.097
Very severe		0.987	0.858-1.136	0.859
Mild & moderate	HDAC2	0.377	0.256-0.554	<0.001
Severe		0.641	0.472-0.871	0.004
Very severe		1.227	0.894-1.685	0.206
Mild & moderate	GSS	0.844	0.685-1.041	0.113
Severe		0.959	0.78-1.178	0.689
Very severe		0.841	0.602-1.176	0.312
Mild & moderate	GSR	1.020	0.958-1.087	0.533
Severe		1.000	0.942-1.061	0.992
Very severe		1.017	0.957-1.08	0.590
Mild & moderate	GPX	1.004	0.988-1.019	0.650
Severe		1.000	0.985-1.016	0.952
Very severe		1.001	0.986-1.016	0.904
Mild & moderate	GCLM	25.300	3.775-169.54	<0.001
Severe		23.720	0.861-653.552	0.061
Very severe		201.767	3.32-12260.417	0.011
Mild & moderate	GCLC	0.498	0.336-0.739	<0.001
Severe		0.828	0.601-1.141	0.249
Very severe		0.849	0.608-1.186	0.337
Mild & moderate	DPP4	0.873	0.452-1.686	0.686
Severe		0.650	0.415-1.019	0.060
Very severe		0.322	0.132-0.785	0.013
Mild & moderate	COX2	1.095	0.941-1.274	0.239
Severe		1.061	0.909-1.239	0.454
Very severe		0.882	0.736-1.058	0.177

Supplementary table S2. Characteristics of the individuals incorporated in the measurement of enzymes involved in systemic inflammation and glutathione (GSH) metabolism mRNA expression in peripheral blood mononuclear cells. Patients diagnosed as having chronic obstructive pulmonary disease (COPD) divided into 3 groups, patients with forced expiratory flow in one second (FEV_1) <50% predicted and fewer than two exacerbations in the previous year, patients with FEV_1 ≥50% predicted and two or more exacerbations in the previous year, and patients with FEV_1 <50% predicted and two or more exacerbations in the previous year according to the Global Initiative for COPD consensus document 2011 (GOLD 2011) [2,3].

	**CD-123**			
**Characteristics**	**1 (n=33)**	**2 (n=2)**	**3 (n=34)**	***p*-value**[**]
Age	67.1 ± 2.2	72.0 ± 0.2	70.1 ± 2.0	0.222
Male	29 (88%)	2 (100%)	33 (97%)	0.310
BMI	25.4 ± 0.9	30.2 ± 5.2	23.4 ± 0.7	0.052
Smoking (pack-years)	37.6 ± 3.7	52.0 ± 2.0	43.2 ± 4.2	0.353
Current Smoker	19 (58%)	1 (50%)	12 (35%)	0.178
Smoking cessation amongst ex-smokers (years ago)	11.4 ± 2.5	4.0 ± 0.0	12.2 ± 2.3	0.322
PEF % predicted	29.1 ± 1.7	48.5 ± 2.5	26.1 ± 1.4	0.039
FEV_1 % predicted	32.8 ± 1.7	50.5 ± 0.5	29.6 ± 1.5	0.025
Absolute decline in FEV_1 % over years (%/year)[*]	2.0 ± 0.3	1.1 ± 0.0	1.6 ± 0.1	0.107
FVC % predicted	49.9 ± 2.2	71.0 ± 7.0	46.1 ± 2.5	0.064
FEV_1/FVC %	53.4 ± 1.8	56.9 ± 4.0	53.0 ± 2.0	0.745

Data are presented as mean ± SEM or n (%).

[*]Annual change in FEV_1 % predicted after the age of 25, assuming FEV_1% was 100% at the age of 25 years.

BMI – body mass index, FEV_1 – forced expiratory volume in one second, FVC – forced vital capacity, PEF - peak expiratory flow.

[**]To test the equality of the data across the study groups, Kruskal-Wallis test and Pearson's chi-square test was applied for numeric and nominal variables, respectively.

References

1. Global Initiative for Chronic Obstructive Lung Disease (GOLD). Global Strategy for the Diagnosis, Management, and Prevention of Chronic Obstructive Pulmonary Disease. 2022 [updated 2022;11 July 2022]. Available from: https://goldcopd.org/

2. Global Initiative for Chronic Obstructive Lung Disease (GOLD). Global Strategy for the Diagnosis, Management, and Prevention of Chronic Obstructive Pulmonary Disease. 2011. Available from: https://goldcopd.org/
3. Lange P, Marott JL, Vestbo J, et al. Prediction of the clinical course of chronic obstructive pulmonary disease, using the new GOLD classification: a study of the general population. Am J Respir Crit Care Med. 2012 Nov 15;186(10):975-81.
